# Student standardized patients versus occupational standardized patients for improving clinical competency among TCM medical students: a 3-year prospective randomized study

**DOI:** 10.1186/s12909-023-04198-0

**Published:** 2023-04-05

**Authors:** Jinhao Zeng, Shuang Liang, Xiaoxu Fu, Jing Guo, Yaolin Bai, Shan Zhou, Quanyu Du, Zhenxing Wang, Xiyu Zhang, Sihan Peng, Lijuan Wen, Wenyuan Li, Bin Li, Han Yang, Yi Zhang

**Affiliations:** 1 Department of Chinese Internal Medicine, Hospital of Chengdu University of Traditional Chinese Medicine, Chengdu, 610072 China; 2 Education Department, Clinical Medical School of Chengdu University of Traditional Chinese Medicine, Chengdu, 610075 China; 3 Clinical Skill Center, Clinical Medical School of Chengdu University of Traditional Chinese Medicine, Chengdu, 610075 China; 4 Sichuan Evidence-Based Medicine Center of Traditional Chinese Medicine, Chengdu, 610072 China

**Keywords:** Standardized patients, Simulation, Clinical competency, TCM education

## Abstract

**Background:**

Standardized patient (SP) simulations are well-recognized patterns for practicing clinical skills and interactions. Our previous study showed that a simulation program using occupational SP for Traditional Chinese Medicine (OSP-TCMs) was efficient, however, a high cost and time-intensive nature have limited its use. TCM postgraduates trained as student SPs (SSP-TCMs) present a potentially cost-effective alternative. The purpose of this study was to examine and determine whether SSP simulation offered more benefits over didactic training alone for improving clinical competency among TCM medical students, and conduct a multifaceted analysis comparing SSP-TCMs and OSP-TCMs.

**Methods:**

This was a prospective, single-blinded, randomized controlled trial. Fourth-year TCM undergraduates were recruited as trainees from the Clinical Medical School, Chengdu University of TCM. Data were collected from September 2018 to December 2020. Trainees were randomly divided into the three following groups: traditional method training group, OSP-TCM training group, and SSP-TCM training group (1:1:1). At the end of a 10-week curriculum, trainees received a two-station examination comprising a systematic online knowledge test and an offline clinical performance examination. Post-training and post-exam questionnaires were administered to collect feedback from these trainees.

**Results:**

Students assigned to the SSP-TCM training and OSP-TCM training groups received favorable marks for the “systematic knowledge test” and “TCM clinical skills” (2018, P^a^=0.018, P^b^=0.042; 2019, P^a^=0.01, P^b^=0.033; 2020, P^a^=0.035, P^b^=0.039) compared to the TM trainees. Additionally, trainees in the intervention groups demonstrated a positive post-training edge in scores of “medical records” (2018, P^a^=0.042, P^b^=0.034; 2019, P^a^=0.032, P^b^=0.042; 2020, P^a^=0.026, P^b^=0.03) and “TCM syndrome differentiation and therapeutic regimen” (2018, P^b^=0.032; 2019, P^a^=0.037, P^b^=0.024; 2020, P^a^=0.036, P^b^=0.043). For the simulation encounter assessment given by SP-TCMs, OSP-TCM trainees and SSP-TCM trainees scored higher than TM trainees (2018, P^a^=0.038, P^b^=0.037; 2019, P^a^=0.024, P^b^=0.022; 2020, P^a^=0.019, P^b^=0.021). For the feedback questionnaires, the students in TM group provided less positive feedback for training efficacy and test performance compared to those in the SSP-TCM and OSP-TCM groups. The trainees responded that the training effect of clinical simulations was similar between the SSP-TCM and OSP-TCM groups. SSP-TCMs were more responsive to unexpected emergencies (P^a^=0.022, P^b^>0.05) and more likely to encourage questioning (P^a^=0.029, P^b^>0.05) but tended to provide implied hints (P^c^=0.015) and utilize medical jargon (P^c^=0.007) as compared to OSP-TCMs.

**Conclusion:**

Simulation training for SSP-TCMs and OSP-TCMs showed great benefits for enhancing clinical competency. SSP-TCM simulation was feasible, practical, and cost-effective, and may serve as an alternative method to OSP-TCM simulation.

**Supplementary Information:**

The online version contains supplementary material available at 10.1186/s12909-023-04198-0.

## Introduction

Simulations often utilize a standardized patient (SP), or a “patient-actor” who has been trained to consistently portray a specific patient role outlined by a script [1] for a real encounter experience for healthcare teaching, practice, evaluation, and research [2]. Patient-based simulation training, particularly featuring opportunities for feedback and repetitive practice [3], is more effective for boosting clinical competence compared to didactic instruction [4–7]. Paula Stillman introduced SP into Chinese medical education in 1991, and it has since been used for instructing and evaluation [8]. Despite progress and preponderance in SP-based simulations in medical education, limited resources and cost of use hinder its utility [9]–[10].

SPs, also known as occupational standardized patients (OSPs), staff and faculty, manikins, or students from higher level cohorts (student standardized patients, SSPs), can be paid actors. SSP was introduced in medical education as early as 1992, whereby third- and fourth-year medical students were recruited to participate as SPs and examiners in the Objective Structured Clinical Examination (OSCE) at the University of Minnesota Medical School [11]. SSPs are usually recruited from higher-level cohorts among student peers. SSP simulation uses student individuals as actors beyond learners in scenarios to minimize cost in combination with other methods, like role-playing. SSP simulation is a complex identity comprising multiple roles, which can help develop and enhance learners’ understanding of professional roles and needs, along with patient-centered healthcare [12]. Additionally, after an SP pilot simulation, postgraduates as participants responded that they gained more experience of interdisciplinary interaction and their confidence in handling clinical work were boosted [13]. Taken together, due to its cost-effectiveness and educational benefits, SSP simulation has been applied in various fields of medical education, including nursing and dental training [6, 14].

Traditional Chinese Medicine (TCM) has its discipline characteristics. At present, teaching modes, including didactic sessions, problem-based learning, and manikin-based skill practice dominate TCM universities in China. These lead to the problems of low enthusiasm and self-directed awareness in learning among the students [15]. Apprenticed approach and bedside teaching of TCM synchronizes the classroom with practice but the model is monotonous (students are more like “visitors” than “participants”) and has no effective evaluation mechanisms. The development of SP in TCM has been relatively late. The Chengdu University of TCM is one of the few TCM universities that conducts SP simulation courses in China. Beginning in 2015, a batch of occupational standardized patients of TCM (OSP-TCMs) were trained and obtained certificates of qualification at the Clinical Skill Center of Clinical Medical School, Chengdu University of TCM. Subsequently, our team conducted a 5-year prospective study and demonstrated the benefits of improving the TCM clinical competency among students who trained with the OSP-TCMs [16]. Although an implementable and efficient OSP-TCM training model has been established, some challenges, like resource constraints of professional social origin SP-TCMs, and the high cost of time and effort, need to be addressed. The present OSP-TCM program does not fully cover these college students.

TCM medical students should be exposed to clinical competency enhancement training as an important part of their summative assessment for pre-clinical and clinical procedures. Since 2018, the Clinical Medical School of Chengdu University of TCM has initiated a student-SP-TCM program and trained a batch of TCM postgraduates as SSP-TCMs. Research on SSP in the field of TCM education has not yet been reported. The main objective of this prospective study was to determine whether SSP-TCM simulation offered benefits over didactic sessions for improving clinical competency among TCM medical students. A multifaceted analysis was also conducted for comparing OSP-TCMs and SSP-TCMs. Our findings are expected to provide a reduced-cost and high-efficiency training mode to enhance clinical competency among students of TCM.

## Methods

### Ethical review

This was a prospective, single-blinded, randomized controlled trial. The study was approved by the ethics committee of Chengdu University of TCM (no. 3801) and was conducted in accordance with the principles of the Declaration of Helsinki. All methods in this study were carried out in accordance with the “standard of undergraduate medical education-Traditional Chinese Medicine” issued by the National Advisory Committee on Traditional Chinese Medicine in Higher Education under the Ministry of Education of China, and with the training guidelines of TCM medical undergraduates of five-year program released by the Chengdu University of TCM. Informed consent was obtained from all participants. At the conclusion of the trial, trainees in the control group were provided with an opportunity to receive systematic training of OSP-TCM simulation.

### Recruitment of SSP-TCMs

18 voluntary first-year postgraduate students were recruited. Students signed appropriate consent forms and participated voluntarily. Voluntary SSP-TCMs should be with physical and mental fitness based on psychological test files and health examination records. Those with acting talent and strong sense of responsibility will be given priority to inclusion. Information on SSP-TCMs were collected including gender, age, scores of the National Entrance Examination for Postgraduate (NEEP), and medical specialty.

### Training and eligibility of SSP-TCMs

Four months prior to the study, the enrolled postgraduate students were trained by several senior SP trainers as student standardized patients of TCM (SSP-TCMs), they received a systematic training course consisting of didactic sessions and skill practice, and self-study sessions. The specific training methods are provided in Supplementary Material 1 and eligibility requirements are shown in our previous study [16]. Finally, 15 SSP-TCMs passed the qualification test assessed by SP instructors and internal medicine specialists (both blinded to the SSP-TCMs), and they later participated the study.

### Trainees

#### Enrollment of trainees

**Fig. 1:**
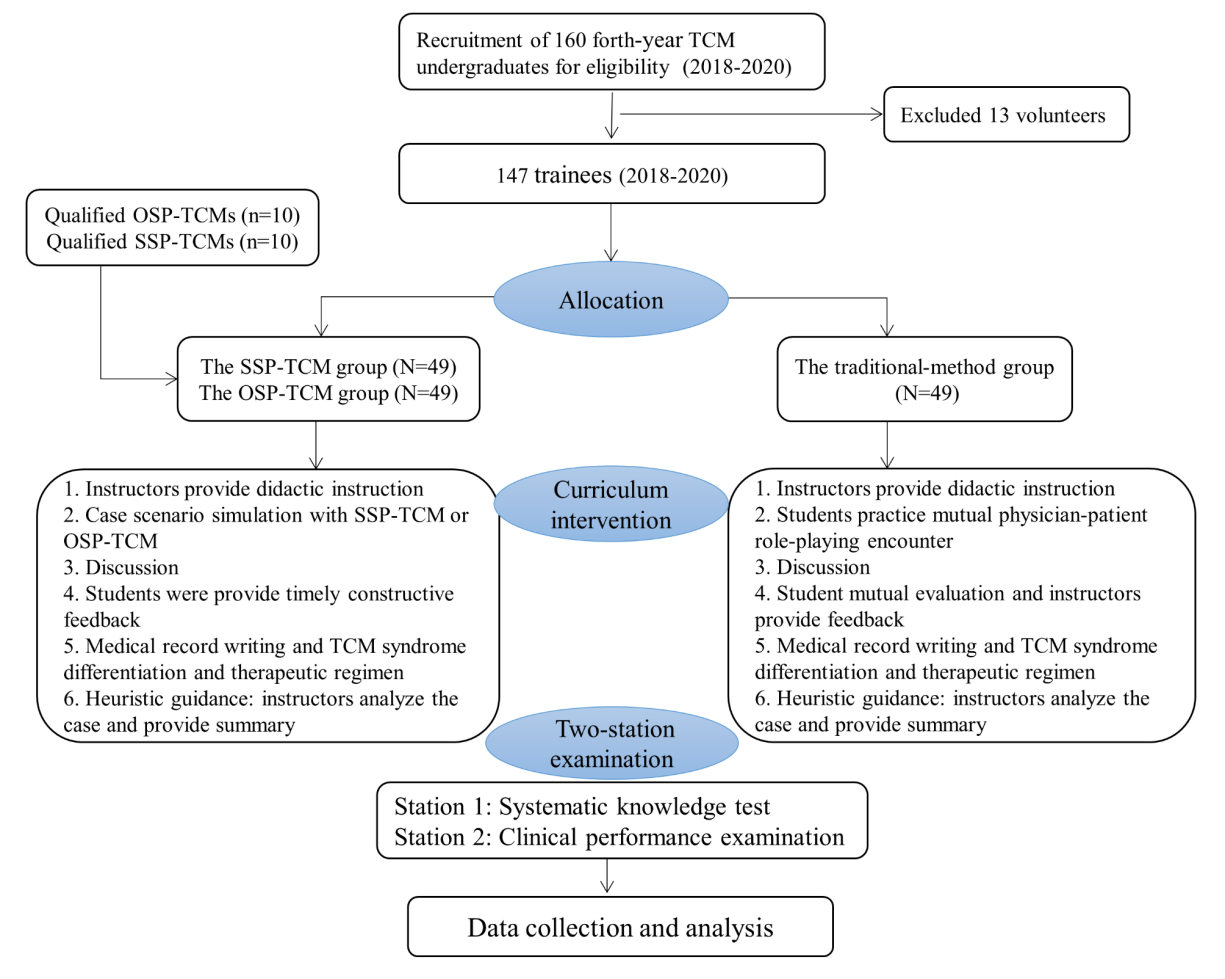
Flow chart of the study protocol

A total of 160 fourth-year TCM undergraduates learning the curriculum of TCM clinical competence enhancement training at the Chengdu University of Traditional Chinese Medicine from September 2018 to December 2020 were offered enrollment. Figure 1 shows the flowchart for enrollment, randomization, intervention and assessment of trainees throughout the trial. Informed consent was obtained for all trainees.

### Inclusion and exclusion criteria

Inclusion criteria for trainees were: (1) Forth-year undergraduates majoring in TCM at the Chengdu University of Traditional Chinese Medicine. (2) Participation was entirely voluntary, and confidentiality agreement was obtained. (3) Trainees passed the examinations of basic courses of Traditional Chinese Medicine and Western Medicine. (4) Trainees in good physical and psychological condition. Exclusion criteria included the following items: (1) Trainees who had received prior formal training as a standardized patient. (2) Trainees who had or being participated in a similar intervention. (3) Trainees who were failure to complied with the confidentiality agreement regarding the curriculum content. (4) Trainees were unwilling or unable to continue training, missing any of the training sessions.

### Randomization and blinding

Using computer-generated randomization, trainees were assigned 1:1:1 to receive either traditional training method (TM group) or OSP-TCM training method (OSP-TCM group) or SSP-TCM training method (SSP-TCM group). An independent investigator carried out the randomization. The assignment and all baseline measures were confidential to the trainees, researchers and study staff involved in this study. Independent study analysts were blinded to collect data during the study period, and the data analysis could not be performed until the data collection was complete.

### Training protocol of curriculum

This was a case-based and group-based curriculum that lasted for 10 weeks with a total of 40 class hours. The curriculum was organized by faculty members as a 4-class hour session comprised of two training clinical case, once a week. The curriculum is taught on simulation clinic at Shi-er-qiao campus of Chengdu University of Traditional Chinese medicine. Training assistants who have signed confidentiality agreements were hired to assist faculty members during the curriculum. Three groups referred to the same course syllabus, utilized the same training clinical cases, experienced the same amount of training time. During the project period, an equal number of SPs were employed, that is, 10 student standardized patients in SSP-TCMs group and 10 occupational standardized patients in OSP-TCMs group. The faculty in the three groups were trained to use the identical rubric (e.g., evaluation of TCM clinical skills, written questions of TCM syndrome differentiation and therapeutic regimen), and completed a norming prior to use. Supplementary Material 1 shows the detailed training flow of the three groups.

### Evaluation of training effectiveness

#### Examination design, confidentiality, and objectivity

All trainees took two-station final examinations– a systematic online knowledge test and an offline clinical performance examination. The exam was scheduled for two days. On the first day, a systematic knowledge test was conducted, whereby all trainees completed 50 objective questions online within 60 min. On the next day, the clinical performance examination lasted 60 min—there was a 20 min patient encounter wherein trainees completed a medical interview, performed a physical examination, and gave medical advice. The evaluation of student performance was immediately completed by OSP-TCMs and TCM specialists using checklists. Subsequently, the trainees had 40 min to write a summary report on the simulation encounter, including a presentation of the medical records, making a diagnosis, and giving TCM syndrome differentiation and therapeutic regimen.

To ensure confidentiality and objectivity, new cases were selected from the clinical case database for testing. To eliminate evaluation bias, another group of senior OSP-TCMs and TCM specialists with no exposure to any trainee served as examination evaluators. All the OSP-TCMs and TCM specialists in the exam received evaluation training to ensure consistency. To maintain high fidelity and consistency in performance, the eligibility of OSP-TCMs was determined again 10 days before the final examination. The clinical performance examinations were conducted in a clinical skills center with three waiting areas and six examination areas, separated by appropriate distance and supervised by video to ensure no communication among the staff and trainees. The entire exam process was audiotaped and videotaped. The items needed for clinical performance examination were prepared in advance, and trainees were not allowed to take anything out of the examination area. For trainees not meeting clinical competency for the assessment, a double-check was conducted—another TCM specialist and senior OSP independently reviewed the video, and both evaluators met to discuss and regrade the trainee’s performance.

### Systematic knowledge test

The systematic knowledge test was a standardized, 50-item, with a total score of 100, multiple choice question online testing. It examined students’ mastery of basic knowledge and clinical skills, along with their cognitive and application abilities. These objective questions covered the following domains: TCM and western medicine, physical examination, auxiliary examination, humanistic care, and doctor-patient communication skills.

### Evaluation of TCM clinical skills

Evaluation for trainees’ proficiency in TCM clinical skills was based on the following dimensions [17]: (1) inspection: 5 points including mental state, facial expression, complexion, physical condition, secretions, and finger venules.; (2) tongue manifestation: 5 points, including changes in the tongue color (pale, pale red, purple, crimson, etc.), form and motility of the tongue body (prickly tongue, spotted tongue, limp wilting tongue, deviated tongue, etc.), and coating (moist or dry, thin or thick, slippery, or slimy fur); (3) listening and smell examination: 5 points, including differentiating abnormal voice and smell (like deep turbid or faint low voice, phlegm rale, fetid mouth odor, etc.); (4) inquiry: 70 points, including basic information (5 points), chief complaints (5 points), present history (30 points), past history (10 points), personal history (10 points), and family history (10 points). Notably, the inquiry domain included some TCM items like “cold and heat”, “condition of sweating”, “Yin-Yang pattern identification”, and “deficiency and excess”, and (5) palpation and pulse diagnosis: 15 points, including three positions and nine indicators of pulsation, along with 28 types of pulse condition. Some TCM-specific symptoms and signs that were not presented in SP-TCMs were orally instructed, or through image presentation (e.g., TCM tongue manifestation), or using specific instruments (e.g., TCM pulse examination). For a more detailed evaluation of the content and related terminology of TCM clinical skills, we referred to the published book [18].

### Scoring of medical records

The scoring for written medical records was based on a standardized, 100-point scale checklist published previously [16] and included general data (3 points), chief complaint (5 points), present history (30 points), past history (10 points), personal history (10 points), family history (6 points), physical examination (20 points), and four examinations of TCM (16 points).

### Scoring for TCM syndrome differentiation and therapeutic regimen

The written records of TCM syndrome differentiation and therapeutic regimen were scored based on a standardized, 100-point scale described previously [16] and included the diagnosis by TCM (6 points), diagnostic evidence for TCM (6 points), diagnosis by Western medicine (6 points), diagnostic evidence for Western medicine (14 points), syndrome type in TCM (10 points), analysis of TCM syndrome differentiation (24 points), method of TCM treatment (8 points), formula (8 points), medicine, their doses and method of administration (14 points), and medical advice (4 points).

### Real-time assessment from SP-TCMs

OSP-TCMs assessed student performances using the Arizona Clinical Interviewing Rating Scale (ACIR) [19]. The rubric contained 20 items on professionalism, communication, and interviewing skills. Each item ranged from 1 to 5 points, with a maximum score of 5. Instructions on using the rubric were given before the exam.

### Post-training and post-exam questionnaires from trainees

To investigate students’ attitudes and perceptions toward the curriculum training, anonymous post-training and post-exam surveys were initiated. The project team developed post-training survey questionnaires using a 4- or 5-point Likert scale based on previously published tools [15, 20−21] Post-training questionnaire comprised 15 items, including “Enhance the quality of medical record”, “Improve TCM thinking ability” and “Satisfy with the teaching effectiveness of the course.” The post-exam questionnaire comprised 5 items, including “Your test performance achieves fidelity rate of your usual performance?”, “Do you think the scenario preparation in examination is authentic?”

### Statistical analysis

Statistical data were analyzed using the SPSS 25.0 software. Data were presented as mean ± standard deviation or percentages. Differences in multi-group comparisons were evaluated using a one-way analysis of variance (ANOVA), followed by the Tukey method for homogeneous data and Dunnett’s T3 method for non-homogeneous data. The comparison of proportion and correlation analyses was conducted using the Chi-square test. p < 0.05 indicated statistical significance.

## Results

### Demographic data of the trainees

Among the 160 trainees, 147 were enrolled in this study (excluding 13 trainees who were ineligible) and none of them were lost to follow-up. In the final analyses, 45 trainees in 2018, 48 in 2019, and 54 in 2020 were included. Descriptive statistics revealed no significant differences in gender and age among the three groups and the three groups across different years (all P > 0.05). At baseline, no significant differences were found for educational background variables for basic courses of TCM and Western medicine among the three groups and the three groups across different years (all P > 0.05). Table 1 shows detailed demographic data of the trainees of the three groups from 2018 to 2020.

**Table 1:** Demographic data of the trainees among the three groups from 2018–2020 (n = 147)

Demographics	SSP-TCM group	OSP-TCM group	Traditional method group	F-value/χ^2^	P-value
2018–2020 (n = 147)					
**Age, mean ± SD**	21.45 ± 0.71	21.43 ± 0.84	21.31 ± 0.87	0.446	0.641
**Gender, n (%)**				0.221	0.895
Female	27 (55.10)	29 (59.18)	27 (55.10)		
Male	22 (44.90)	20 (40.82)	22 (44.90)		
**Basic courses of Traditional Chinese Medicine, mean ± SD**					
Fundamental theory of TCM	72.16 ± 2.94	72.22 ± 3.71	72.06 ± 3.26	0.030	0.970
Chinese materia medica	76.35 ± 4.65	76.20 ± 3.28	75.80 ± 3.59	0.265	0.767
Diagnostics of TCM	75.67 ± 2.30	75.53 ± 2.14	75.98 ± 1.80	0.591	0.555
Formulaology of TCM	75.76 ± 2.75	75.78 ± 2.34	76.22 ± 1.81	0.635	0.531
**Basic courses of Western Medicine, mean ± SD**					
Anatomy	75.65 ± 2.31	75.92 ± 2.77	75.45 ± 3.80	0.296	0.744
Physiology	71.92 ± 2.47	71.94 ± 2.98	72.31 ± 3.32	0.269	0.764
Pathology	75.08 ± 2.75	75.08 ± 2.75	74.49 ± 4.36	0.506	0.604
Medical biology	74.04 ± 2.58	74.10 ± 2.76	74.45 ± 2.65	0.334	0.716
Diagnostics of western medicine	74.29 ± 2.38	74.06 ± 2.80	74.47 ± 2.93	0.278	0.758
**2018 (n = 45)**					
**Age, mean ± SD**	21.47 ± 0.64	21.40 ± 0.74	21.27 ± 1.16	0.202	0.817
**Gender, n (%)**				0.180	0.914
Female	9 (60.00)	8 (53.33)	8 (53.33)		
Male	6 (40.00)	7 (46.67)	7 (46.67)		
**Basic courses of Traditional Chinese Medicine, mean ± SD**					
Fundamental theory of TCM	72.20 ± 3.12	72.33 ± 3.92	72.67 ± 3.85	0.078	0.925
Chinese materia medica	75.60 ± 5.04	74.73 ± 3.43	75.47 ± 2.77	0.218	0.805
Diagnostics of TCM	77.27 ± 1.58	76.40 ± 2.17	75.87 ± 1.41	2.452	0.098
Formulaology of TCM	76.67 ± 2.26	77.13 ± 2.36	76.13 ± 1.77	0.818	0.448
**Basic courses of Western Medicine, mean ± SD**					
Anatomy	75.53 ± 2.39	75.53 ± 2.42	75.80 ± 2.31	0.063	0.939
Physiology	71.67 ± 2.06	70.33 ± 1.40	70.87 ± 2.17	1.862	0.168
Pathology	75.53 ± 2.17	74.73 ± 2.49	74.13 ± 2.59	1.261	0.294
Medical biology	74.60 ± 3.11	74.20 ± 3.05	75.13 ± 2.45	0.395	0.676
Diagnostics of western medicine	74.80 ± 3.19	74.93 ± 2.71	74.07 ± 2.34	0.426	0.656
**2019 (n = 48)**					
**Age, mean ± SD**	21.50 ± 0.63	21.44 ± 0.96	21.31 ± 0.70	0.240	0.788
**Gender, n (%)**				0.171	0.918
Female	9 (56.25)	10 (62.50)	9 (56.25)		
Male	7 (43.75)	6 (37.50)	7 (43.75)		
**Basic courses of Traditional Chinese Medicine, mean ± SD**					
Fundamental theory of TCM	71.56 ± 2.34	71.81 ± 2.76	71.81 ± 3.76	0.037	0.964
Chinese materia medica	74.88 ± 5.03	75.69 ± 2.92	75.31 ± 4.66	0.143	0.867
Diagnostics of TCM	76.38 ± 1.36	76.44 ± 1.75	77.00 ± 1.16	0.911	0.410
Formulaology of TCM	76.63 ± 2.10	76.63 ± 1.71	77.13 ± 1.15	0.464	0.632
**Basic courses of Western Medicine, mean ± SD**					
Anatomy	75.69 ± 1.92	75.56 ± 2.58	74.75 ± 2.02	0.862	0.429
Physiology	71.94 ± 1.57	72.44 ± 3.74	72.31 ± 1.89	0.162	0.851
Pathology	75.75 ± 2.49	75.94 ± 2.79	75.13 ± 3.18	0.360	0.699
Medical biology	74.06 ± 2.57	74.56 ± 2.50	74.19 ± 2.88	0.154	0.858
Diagnostics of western medicine	74.31 ± 1.89	73.81 ± 2.29	75.50 ± 2.94	2.066	0.139
**2020(n = 54)**					
**Age, mean ± SD**	21.39 ± 0.85	21.44 ± 0.86	21.33 ± 0.77	0.082	0.922
**Gender, n (%)**				0.450	0.799
Female	10 (55.56)	11 (61.11)	9 (50.00)		
Male	8 (44.44)	7 (38.89)	9 (50.00)		
**Basic courses of Traditional Chinese Medicine, mean ± SD**					
Fundamental theory of TCM	72.67 ± 3.31	72.50 ± 4.38	71.78 ± 3.23	0.297	0.744
Chinese materia medica	78.28 ± 3.38	77.89 ± 2.83	76.50 ± 3.19	1.598	0.212
Diagnostics of TCM	73.72 ± 2.11	74.00 ± 1.57	75.17 ± 2.15	2.750	0.073
Formulaology of TCM	74.22 ± 3.06	73.89 ± 1.49	75.50 ± 2.04	2.483	0.094
**Basic courses of Western Medicine, mean ± SD**					
Anatomy	75.72 ± 2.68	75.56 ± 3.20	75.78 ± 5.67	0.236	0.790
Physiology	72.11 ± 3.39	72.83 ± 2.77	73.50 ± 4.55	0.653	0.525
Pathology	74.11 ± 3.12	74.61 ± 2.89	74.22 ± 6.22	0.066	0.937
Medical biology	73.56 ± 2.12	73.61 ± 2.81	74.11 ± 2.63	0.262	0.771
Diagnostics of western medicine	73.83 ± 2.01	73.56 ± 3.24	73.89 ± 3.25	0.069	0.934

### Scores for the systematic knowledge test

**Fig. 2:**
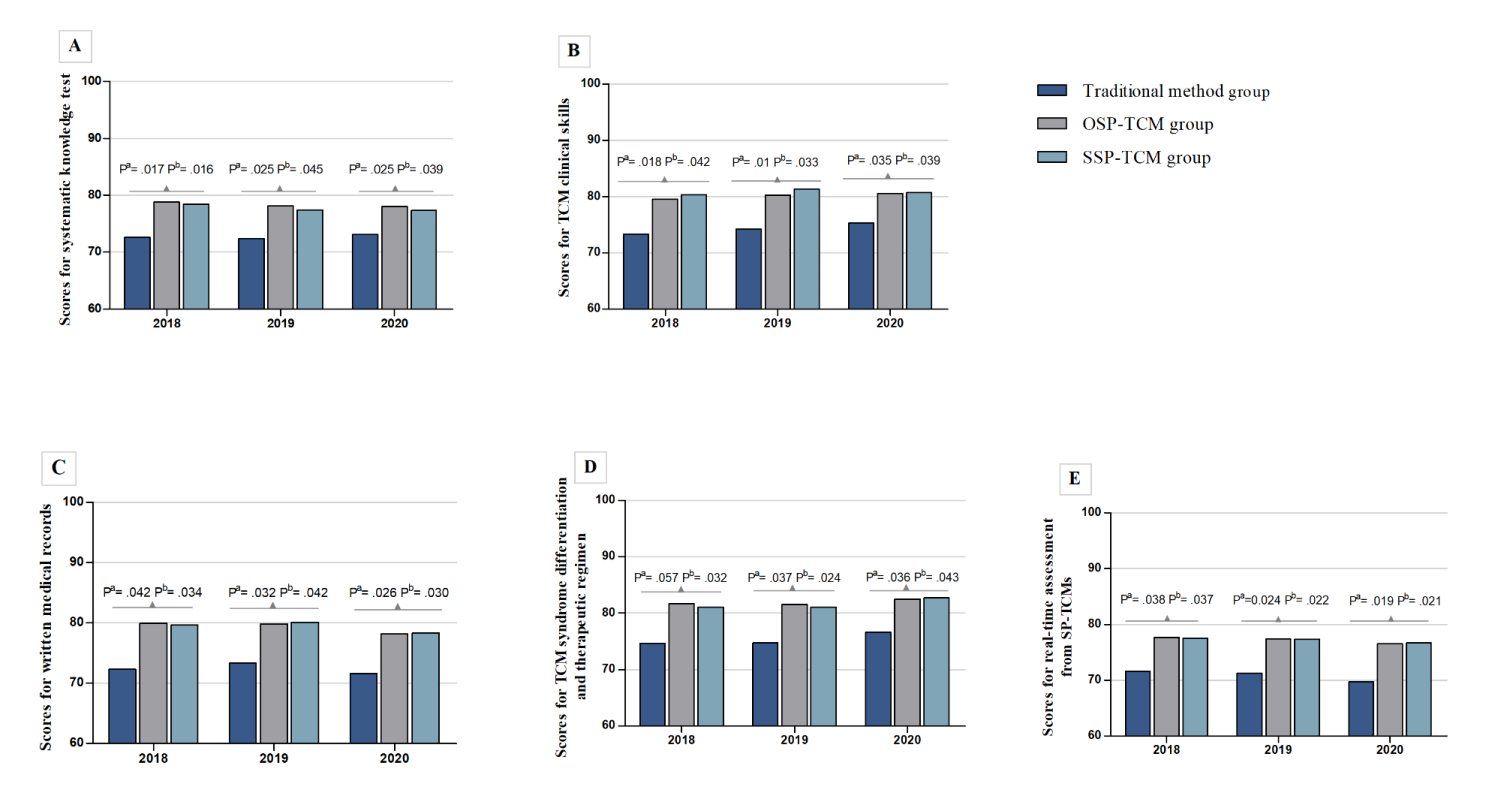
The results of standardized examination among the TM group, OSP-TCM group and SSP-TCM group (2018–2020). Graphs show 3-group comparisons including (**A**) scores for systematic knowledge test, (**B**) scores for TCM clinical skills, (**C**) scores for written medical records, (**D**) scores for TCM syndrome differentiation and therapeutic regimen, and (**E**) sores for real-time assessment from SP-TCMs. Note: 2-way contrasts are depicted by the gray lines and P values ^a^SSP-TCM group vs. TM group, ^b^OSP-TCM group vs. TM group, ^c^SSP-TCM group vs. OSP-TCM group

The objective questions set in advance on the computer system were used for intelligence grading in the systematic knowledge test, and the examinees’ report cards were extracted. The scores of systematic knowledge, and the proportion of trainees with scores above 80, were distinctly higher between the OSP-TCM and SSP-TCM groups but not in the TM group. The results of one-way ANOVA for comparing the differences among the three groups (2018/19/20, F = 4.484/3.338/3.386, P = 0.017/0.044/0.042), along with the post-hoc analysis suggested a statistically significant difference in the overall grading between the SSP-TCM and TM groups (2018/19/20, P^a^=0.028/0.022/0.020), and between the OSP-TCM and TM groups (2018/19/20, P^b^=0.042/0.045/0.043). However, no statistical differences were observed between the OSP-TCM and SSP-TCM groups (2018/19/20, all P^c^ > 0.05). The results are shown in Fig. 2A (Note: a = SSP-TCM group vs. TM group, b = OSP-TCM group vs. TM group, c = SSP-TCM group vs. OSP-TCM group).

### Scores for TCM clinical skills

For the application of TCM clinical skills, results of one-way ANOVA for comparing the differences among the three groups (2018/19/20, F = 4.836/5.434/4.310, P = 0.013/0.008/0.019) and post-hoc analysis showed that trainees in both SSP-TCM and OSP-TCM groups exhibited significantly greater improvements relative to those in the TM group (2018/19/20, P^a^=0.018/0.01/0.035; P^b^=0.042/0.033/0.039). During the entire study, no significant differences were found between the SSP-TCM and OSP-TCM groups (2018/19/20, all P^c^ > 0.05). These results suggested that performing case scenario simulation by OSP-TCMs or SSP-TCMs offered an advantage over traditional didactic training. The outcome of TCM clinical skills application among the three groups is shown in Fig. 2B.

### Scores for written medical records

After the clinical performance examination, the quality of written medical records was scored. The results of one-way ANOVA for comparing the differences among the three groups (2018/19/20, F = 4.352/4.359/4.700, P = 0.019/0.019/0.013) and post-hoc analysis revealed that trainees of both the OSP-TCM and SSP-TCM groups had more favorable scores than those in TM group, with a statistically significant difference in the overall grading between the SSP-TCM and TM groups (2018/19/20, P^a^=0.042/0.032/0.026), and between the OSP-TCM and TM groups (2018/19/20, P^b^=0.034/0.042/0.030). Moreover, when comparing the scores between the SSP-TCM and OSP-TCM groups, no statistical differences were observed (2018/19/20, all P^c^ > 0.05). The scores of written medical records in the three groups are shown in Fig. 2C.

### Scores for TCM syndrome differentiation and therapeutic regimen

For TCM syndrome differentiation and therapeutic regimen, the results of one-way ANOVA for comparing the differences among the three groups (2018/19/20, F = 4.171/4.681/4.223, P = 0.022/0.014/0.020) and post-hoc analysis revealed that trainees assigned to SSP-TCM and OSP-TCM groups scored higher than those assigned to TM group (2018/19/20, P^a^=0.057/0.037/0.036; P^b^=0.032/0.024/0.043). A similar trend was noted in the first year, i.e., in 2018, although no statistical difference was found between the SSP-TCM and TM groups (2018, P^a^=0.057). Additionally, mean scores for TCM syndrome differentiation and therapeutic regimen were similar between the SSP-TCM and OSP-TCM groups (2018/19/20, all P^c^ > 0.05). The results are presented in Fig. 2D.

### Scores for real-time assessment from SP-TCMs

For encounter assessment given by SP-TCMs, the results of one-way ANOVA for comparing the differences among the three groups (2018/19/20, F = 4.421/5.035/5.169, P = 0.018/0.011/0.009) and post-hoc analysis showed that OSP-TCM and SSP-TCM trainees achieved a similar performance score, and no significant differences were found (2018/19/20, all P^c^ > 0.05). In contrast, TM trainees scored lower than OSP-TCM trainees or SSP-TCM trainees in the simulation encounter (2018/19/20, P^a^=0.038/0.024/0.019; P^b^=0.037/0.022/0.021). In our secondary analyses using the ACIR rubric for assessing the performance of OSP-TCM and SSP-TCM trainees, the latter tended to encourage patients to ask questions and were more likely to use “citation and verification” during the clinical encounter. By contrast, OSP-TCM trainees were able to avoid relevant medical jargon, were concerned more about the impact of the disease and the patient’s expectations, and always checked the patient’s understanding. Real-time assessment for performance examination from SP-TCMs is shown in Fig. 2E.

### Post-training feedback analysis

Table 2 shows the results of post-training feedback in the three groups. Trainees in both SSP-TCM and OSP-TCM groups reported that they achieved greater improvements relative to the TM group in “the quality of medical record” (χ^2^ = 10.584, P^a^=0.032; χ^2^ = 10.04, P^b^=0.04), “TCM thinking ability” (χ^2^ = 10.781, P^a^=0.032; χ^2^ = 10.585, P^b^=0.029), “physician-patient interpersonal and communication skills” (χ^2^ = 12.532, P^a^=0.014; χ^2^ = 9.893, P^b^=0.042), and “the confidence in handling clinical work” (χ^2^ = 10.667, P^a^=0.031; χ^2^ = 9.840, P^b^=0.043). Moreover, trainees in SSP-TCM and OSP-TCM groups reported a higher satisfaction index for the training course (χ^2^ = 11.056, P^a^=0.026; χ^2^ = 9.787, P^b^=0.044) with a greater improvement in “interest in learning TCM” following the training (χ^2^ = 9.642, P^a^=0.047; χ^2^ = 10.094, P^b^=0.039) compared to TM controls. They became more proficient in inquiry skills for obtaining the TCM medical history (χ^2^ = 11.903, P^a^=0.018; χ^2^ = 10.543, P^b^=0.032).

**Table 2:** Post-training feedback on curriculum from trainees in three groups (n = 147)

Items	SSP-TCM group (n, %)					OSP-TCM group (n, %)					Traditional method group (n, %)					P-value (χ^2^)^a^	P-value (χ^2^)^b^	P-value (χ^2^)^c^
	Strongly agree	Agree	Neutral	Disagree	Strongly disagree	Strongly agree	Agree	Neutral	Disagree	Strongly disagree	Strongly agree	Agree	Neutral	Disagree	Strongly disagree			
Improve your interest in learning TCM.	8(16.33)	20(40.82)	11(22.45)	7(14.29)	3(6.12)	9(18.37)	18(36.73)	13(26.53)	6(12.24)	3(6.12)	5(10.20)	11(22.45)	10(20.41)	11(22.45)	12(24.49)	0.047(9.642)	0.039(10.094)	0.982(0.408)
Satisfy with the training effectiveness of the course.	13(26.53)	15(30.61)	14(28.57)	6(12.24)	1(2.04)	12(24.49)	16(32.65)	14(28.57)	5(10.20)	2(4.08)	5(10.20)	9(18.37)	18(36.73)	10(20.41)	7(14.29)	0.026(11.056)	0.044(9.787)	0.974(0.497)
Consolidate fundamental theories previously learned.	11 (24.00)	14(28.57)	13(26.53)	7(14.29)	4(8.16)	9(18.37)	12(24.49)	14(28.57)	11(22.45)	3(6.12)	5(10.20)	10(20.41)	17(34.69)	11(22.45)	6(12.24)	0.315(4.739)	0.624(2.615)	0.840(1.423)
Accelerate proficiency in collection of TCM medical history.	12(24.49)	16(32.65)	13(26.53)	8(16.33)	0(0.00)	15(30.61)	13(26.53)	13(26.53)	7(14.29)	1(2.04)	6(12.24)	9(18.37)	15(30.61)	12(24.49)	7(14.29)	0.018(11.903)	0.032(10.543)	0.789(1.710)
Enhance the quality of medical record.	15(30.61)	12(24.49)	18(30)	2(4.08)	2(4.08)	13(26.53)	18(36.73)	13(26.53)	4(8.16)	1(2.04)	6(12.24)	11(22.45)	17(34.69)	10(20.41)	5(10.20)	0.032(10.548)	0.040(10.040)	0.533(3.149)
Improve TCM thinking ability.	17(34.69)	14(28.57)	13(26.53)	4(8.16)	1(2.04)	12(24.49)	20(40.82)	13(26.53)	2(4.08)	2(4.08)	7(14.29)	12(24.49)	14(28.57)	9(18.37)	7(14.29)	0.032(10.781)	0.029(10.585)	0.571(2.921)
Improve physician-patient interpersonal and communication skills.	12(24.49)	17(34.69	14(28.57)	5(10.20)	1(2.04)	10(20.41)	19(38.78)	18(36.73)	6(12.24)	2(4.08)	5(10.20)	11(22.45)	13(26.53)	12(24.49)	8(16.33)	0.014(12.532)	0.042(9.893)	0.928(0.874)
Boost your confidence in handling clinical work.	11(22.45)	20(40.82)	13(26.53)	3(6.12)	2(4.08)	10(20.41)	18(36.73)	16(32.65)	3(6.12)	2(4.08)	4(8.16)	15(30.61)	13(26.53)	12(24.49)	5(10.20)	0.031(10.667)	0.043(9.840)	0.977(0.463)
Get any implied hint in your simulations.	13(26.53)	17(34.69)	12(24.49)	5(10.20)	2(4.08)	6(12.24)	10(20.41)	11(22.45)	17(34.69)	5(10.20)	5(10.20)	11(22.45)	14(28.57)	10(20.41)	9(18.37)	0.025(11.116)	0.485(3.456)	0.015(12.268)
Cooperate with clinical physical examination.	14(28.57)	19(38.78)	12(24.49)	3(6.12)	1(2.04)	10(20.41)	19(38.78)	17(34.69)	3(6.12)	0(0.00)	6(12.24)	12(24.49)	18(36.73)	9(18.37)	4(8.16)	0.029(10.781)	0.048(9.609)	0.639(2.529)
The patient has the ability to respond to emergencies	12(24.49)	18(36.73)	12(24.49)	5(10.20)	2(4.08)	9(18.37)	20(40.82)	17(34.69)	5(10.20)	4(8.16)	6(12.24)	9(18.37)	14(28.57)	13(26.53)	7(14.29)	0.022(11.487)	0.058(9.121)	0.787(1.722)
Encourage students to ask questions.	14(28.57)	19(38.78)	11(22.45)	3(6.12)	2(4.08)	10(20.41)	20(40.82)	13(26.53)	5(10.20)	1(2.04)	7(14.29)	10(20.41)	19(38.78)	8(16.33)	5(10.20)	0.029(10.818)	0.080(8.347)	0.792(1.692)
Repeat and check the students’ understanding.	5(10.20)	10(20.41)	15(30.61)	12(24.49)	7(14.29)	12(24.49)	18(36.73)	9(18.37)	8(16.33)	2(4.08)	12(24.49)	15(30.61)	16(32.65)	5(10.20)	1(2.04)	0.023(11.297)	0.516(3.258)	0.036(10.246)
The “patient” utilizes medical jargon in simulations.	15(30.61)	13(26.53)	13(26.53)	6(12.24)	2(4.08)	4(8.16)	8(16.33)	16(32.65)	13(26.53)	8(16.33)	7(14.29)	8(16.33)	16(32.65)	8(16.33)	10(20.41)	0.040(10.029)	0.693(2.231)	0.007(14.048)
The patient’s performance is exaggerated and emotional.	6(12.24)	18(36.73)	10(20.41)	7(14.29)	8(16.33)	3(6.12)	6(12.24)	16 (32.65)	11(22.45)	13(26.53)	3(6.12)	7(14.29)	16(32.65)	16(32.65)	7(14.29)	0.029(10.813)	0.591(2.803)	0.033(10.464)

^a^SSP-TCM group versus TM group at post-training. ^b^OSP-TCM group versus TM group at post-training. ^c^SSP-TCM group versus OSP-TCM group at post-training. Data are represented in the (n, %). P-values were calculated using Chi-square test

Interestingly, SSP-TCMs received more positive comments from trainees than OSP-TCMs in specific aspects. They responded flexibly to unexpected emergencies (χ^2^ = 11.487, P^a^=0.022; χ^2^ = 9.121, P^b^>0.05), actively cooperated with clinical physical examination (χ^2^ = 10.781, P^a^=0.029), and encouraged the students to ask questions (χ^2^ = 10.818, P^a^=0.029; χ^2^ = 8.347, P^b^>0.05). However, a majority of trainees reported that SSP-TCMs were more likely to provide implied hints (30/49, 61.22%, χ^2^ = 12.268, P^c^=0.015) and utilize medical jargon (28/49, 57.14%, χ^2^ = 14.048, P^c^=0.007) compared to OSP-TCMs. By contrast, more trainees considered that OSP-TCMs would value and check their understanding (χ^2^ = 10.246, P^c^=0.036) and they possessed a slightly higher proficiency in simulation ability (they were less exaggerated and emotional) (χ^2^ = 10.464, P^c^=0.033) than SSP-TCMs.

### Post-examination feedback analysis

Table 3 summarizes the student feedback on examination. The results of post-examination questionnaires revealed that 38.78% of SSP-TCM trainees, 34.69% of OSP-TCM trainees and 12.24% of TM trainees considered their test performances are near or even equal to usual performance, respectively. Intriguingly, more TM trainees than either SSP-TCM trainees or OSP-TCM trainees thought the “patient” in examination act like a real patient (very likely, 36.73% TM vs. 12.24% SSP-TCM vs. 14.29% OSP-TCM). A similar trend was observed in terms of the authenticity of clinical scenario settings, with more TM trainees deemed that the clinical scenario settings was immersive (very much, 42.86% TM vs. 20.41% SSP-TCM vs. 26.53% OSP-TCM). Encounter time was deemed “adequate” by 51.02% of SSP-TCM trainees vs. 46.94% of OSP-TCM trainees vs. 32.65% of TM trainees, while none reported “too long” except for 3 SSP-TCM trainees. A majority of SSP-TCM trainees and OSP-TCM trainees reported that video recording did not (32.65% SSP-TCM trainees vs. 34.69% OSP-TCM trainees) or only slightly (38.78% vs. 28.57%) interfered with their performance. TM trainees, by contrast, believed they were more likely to be interfered by video recording (42.86% very much, 36.73% slightly).

**Table 3:** Post-examination feedback from trainees in three groups (n = 147)

	SSP-TCM group (n, %)				OSP-TCM group (n, %)				TM group (n, %)				P-value(χ^2^)^a^	P-value(χ^2^)^b^	P-value(χ^2^)^c^
Your test performance achieves what fidelity rate of your usual performance?	>99% 19(38.78)	71–99% 15(30.61)	40–70% 12(24.49)	<40% 3(6.12)	>99% 17(34.69)	71–99% 19(38.78)	40–70% 11(22.45)	<40% 2(4.08)	>99% 6(12.24)	71–99% 20(40.82)	40–70% 19(38.78)	<40% 4(8.16)	0.027(9.198)	0.044(8.087)	0.843(0.825)
Do you think the “patient” in examination act like a real patient?	Yes, very likely 6(12.24)	Acceptable 16(32.65)	No, unlikely 19(38.78)	No opinion 8(16.33)	Yes, very likely 7(14.29)	Acceptable 15(30.61)	No, unlikely 15 (30.61)	No opinion 12(24.49)	Yes, very likely 18(36.73)	Acceptable 15 (30.61)	No, unlikely 11(22.45)	No opinion 5(10.20)	0.031(8.858)	0.040(8.338)	0.710(1.380)
Do you think the scenario preparation is authentic in examination?	Yes, very much 10(20.41)	Yes, but slightly 17(34.69)	Not at all 17(34.69)	No opinion 5(10.20)	Yes, very much 13(26.53)	Yes, but slightly 17(34.69)	Not at all 17(34.69)	No opinion 2(4.08)	Yes, very much 21(42.86)	Yes, but slightly 14(28.57)	Not at all 7(14.29)	No opinion 7(14.29)	0.034(8.694)	0.028(9.117)	0.642(1.677)
The time length for the patient encounter in examination is?	Adequate 25(51.02)	Too long 3(6.12)	Too short 13 (26.53)	No opinion 8(16.33)	Adequate 23(46.94)	Too long 0(0.00)	Too short 21(42.86)	No opinion 5(10.20)	Adequate 16(32.65)	Too long 0(0.00)	Too short 19(38.78)	No opinion 14(28.57)	0.052(7.737)	0.060(5.620)	0.129(5.658)
Dose the video recording in examination interferes with your performance?	Yes, very much 9(18.37)	Yes, but slightly 19(38.78)	Not at all 16(32.65)	No opinion 5(10.20)	Yes, very much 12(24.49)	Yes, but slightly 14(28.57)	Not at all 17(34.69)	No opinion 6(12.24)	Yes, very much 21(42.86)	Yes, but slightly 18(36.73)	Not at all 6(12.24)	No opinion 4(8.16)	0.024(9.484)	0.035(8.615)	0.727(1.307)

^a^SSP-TCM group versus TM group at post-examination. ^b^OSP-TCM group versus TM group at post-examination. ^c^SSP-TCM group versus OSP-TCM group at post-examination. Data are represented in the (n, %). P-values were calculated using Chi-square test

## Discussion

In medical education, given patient safety and rights, patients as a core element in actual clinical settings are regularly absent from pre-clinical training. SPs as salaried persons who portray patients’ symptoms and signs consistently have emerged with the demand. [22] When needed, they cooperate with props and makeup techniques to construct an extra credible and secure learning context for clinical interactions with medical students. [23] In this educational setting that emphasizes early clinical encounters and workplace-based assessment, allowing trainees to safely make mistakes and capture ongoing data will facilitate the identification and addressing of learning needs, thus bridging the gap between medical theory and clinical practice [24]–[25].

This study showed substantial differences between SSP-TCM trainees and TM trainees and between OSP-TCM trainees and TM trainees for evaluation parameters in the systematic knowledge test and clinical competence examination. The results corresponded to those of our previous occupational SP program, suggesting that TCM-tailored SP training was more effective than traditional didactic training. [16] While the average score of “TCM syndrome differentiation and therapeutic regimen” of SSP-TCM trainees was higher relative to TM trainees in 2018, the results did not show a statistically significant difference. A major contributor may be the heterogeneity in the system knowledge and case analysis abilities among students. Our data demonstrated that the clinical scenario simulation training incorporated with either SSPs or OSPs was efficient and worth repeating.

In this study, SSP-TCMs were selected from among graduate students majoring in internal TCM medicine and met the general accuracy and consistency requirements for SSPs after brief training. In comparison to OSPs, SSPs showed faster acceptance, shorter training time, and lower training difficulty. [26] In addition to the above-mentioned advantages, SSP-TCMs had outstanding simulation competence due to their high self-efficacy, ensuring the acquisition of knowledge and skills. They were also involved in the development of script renewal. Similar results were reported by other teams [27]–[28]. In comparison, OSP training required self-contained coaching teams and specific hospital teaching observation, tough on a low budget. [14, 29] We, therefore, believe that the SSP-TCM program is potentially a cost-effective alternative.

Regarding feedback on curriculum, SSP-TCM trainees demonstrated significantly improved proficiency in inquiry skills and TCM thinking, especially physician-patient communication skills, proficiency in recording TCM medical history, the quality of medical records, and confidence in handling clinical work. As students, SSP-TCMs participated in the curricular training in a more interactive education format with specific objectives and valued the experience of portraying patients. They also acquired empathy from the perspective of patients. [23] Some features of SSPs may have contributed to these findings, and consequently, they can grant targeted and valuable feedback on communication delivery and student empathy to trainees.

As an emerging medical education tool, SP combined with feedback and reflection has formative benefits for improvement in clinical competence among medical trainees [30]–[31]. Through training, the combination of role-playing with feedback, repetitive practice, and faculty mentorship would improve session communication skills as a core competency [32]. Moreover, SSP-TCMs were more responsive to unexpected emergencies, showed active cooperation with clinical physical examination, and encouraged trainees to ask questions. Relative to OSP-TCMs, however, SSP-TCMs were more likely to offer implied guidelines and utilize medical jargon. This is in part due to the medical background of the SSP-TCMs and the realistic doctor role of the postgraduate SPs undergoing “Standardized Training for Residents” which may interfere with their simulation presentation.

### Limitations

Enlisting graduate students with certain medical knowledge as SSP for teaching has many advantages in medical education. However, some limitations of this study warrant consideration. First, this study was performed in a single institution, and the potential systematic differences in the characteristics of trainees implied reduced external generalizability of the findings. Second, the medical background of SSP-TCMs would inevitably affect their performance in a pretend scenario. For instance, the use of medical terms in role-play simulations is frequent in the initial imitation but gradually decreases as they become more proficient at what they do. Third, the personnel has limited scope and quick turn-round. Graduate students’ mobility is so high that retraining a new batch of SSPs to cope with the graduation of the previous SSPs is required. Finally, the inevitable age limitations of the graduate students make the imitation of age-appropriate standardized patients difficult. The lack of diverse characters (like the elderly, infants, and pregnant women) reduces extrapolation to real-life scenarios. Therefore, well-trained occupational SPs need to be recruited to address these shortcomings.

## Conclusion

The cultivation of clinical competency is an important pillar to be integrated into the entire TCM curriculum, and educators now face the difficulty of creating a learning environment that cultivates clinical practice skills in a highly efficient and productive manner. Simulation training with the SSP-TCMs and OSP-TCMs evidenced great benefits for enhancing clinical competency. Relative to OSP-TCMs simulation, SSP-TCM simulation was feasible, practical, and cost-effective. SSP-TCM simulation could serve as an alternative method for practicing, learning, evaluating, and testing. In the future, we want to assess the effects and differences of diverse types of SPs, like virtual standardized patients, for improvement in clinical competence among TCM medical students.

## Electronic supplementary material

Below is the link to the electronic supplementary material.

Supplementary Material 1

Supplementary Material 2

Supplementary Material 3

Supplementary Material 4

Supplementary Material 5

Supplementary Material 6

Supplementary Material 7

## Data Availability

The data generated or analyzed during this study are available in this published article and its supplementary information files.
