# Supplementary material for: Student standardized patients versus occupational standardized patients for improving clinical competency among TCM medical students: a 3-year prospective randomized study
Source: BMC Med Educ. 2023 Apr 5;23:216. doi: 10.1186/s12909-023-04198-0 (PMC10074708; doi:10.1186/s12909-023-04198-0)
Supplement: Supplementary file 1 — Supplementary Material 1 [file 12909_2023_4198_MOESM1_ESM.docx]

**Supplementary Material 1. Detailed training flow of the three groups.**

**(1) Curriculum detailed training flow of the three groups.**

The curriculum teaching flow of the SSP-TCM group and the OSP-TCM group were the same, and OSPs and SSPs were incorporated into the classroom with a more interactive format. The [identical](C:/Program%20Files%20(x86)/Youdao/Dict/8.9.6.0/resultui/html/index.html#/javascript:;) cases as the TM group were selected and followed as these steps: 1) Teachers provide theoretical instruction that review foundational content concerning the disease 2) Case scenario simulation with SSP-TCM/OSP-TCM: Each SP clinical encounter include a doctor-patient interview, followed by a presentation of the patient’s complaint and treatment options. Then it involved a brief additional encounter. Patient information, such as personal data, disease course, pertinent history and physical findings, were presented upon the script of clinical cases. Some TCM- specific symptoms and signs were given orally, or through image presentation such as TCM tongue manifestation, or using specific instruments such as TCM pulse-taking. 3) Discussion and questions-answer: students were expected to discuss suspected diagnosis and present them with specific treatment recommendations. 4) SSP-TCMs/OSP-TCMs provide timely constructive feedback on their execution (e.g., pointing out the insufficiency, giving their advice and correct demonstration.), they initiate a discussion and exchange ideas, and then they repeat the clinical encounter to improve performance (teachers can assist and instruct during the period.). 5) Team written record: Students complete the medical record writing and TCM syndrome differentiation and therapeutic regimen. 6) Heuristic guidance: teachers conduct professional analysis and provide summary of the case. For both training environments, student questions were answered during the education.

Traditional Method Group (TM Group) carried out traditional collective classroom pattern to conduct TCM internal medicine as follows: 1) Teachers provide theoretical instruction that review foundational content concerning the disease 2) Students practice mutual physician-patient role-playing encounter in pairs. 3) Discussion and questions-answer: After the demonstration, students were expected to commented on the advantages and disadvantages of diagnosis and measures, and put forward their suggestions. 4) Student mutual evaluation and teachers comment on the student-paired simulated performance. 5) Team written record: Students complete classroom exercises, such as the medical record writing and TCM syndrome differentiation and therapeutic regimen. 6) Heuristic guidance: teachers supplement and correct the answers, point out the key and difficult points, and summarize the case.

**(2) Training of SP-TCMs and** **script** **renewal**

First of all, it is necessary to select the case materials of common diseases in TCM internal medicine training, and the script writing work should be completed jointly by the instructors and SP-TCMs. The script content includes the simulation of the scenario, medical history, diagnosis of TCM and western medicine, examination, treatment, follow-up visit, improvement, etc. The SPs language in the script must be concise and easy to understand, try to be colloquial, avoid the use of medical terms, and add appropriate expressions and action requirements. And should also cooperate with props and makeup techniques when necessary. The plot would be discussed by instructor’ team followed by a semi-structured interview with SP-TCMs reported open-ended questions, and then the first draft was written by instructors.

The script was sent to the students to familiarize them with the plot for 7 days, during which the students were organized to observe the typical cases for 2 times to feel the emotion, language and body language. A senior SP trainer adopted multimodality educational intervention consisted of didactic sessions and skills practice using standardized training materials (including clinical clerkships training sessions), which included approximately 4 class hours of theoretical teaching and 16 class hours of clinical field simulation training. During a four-week clerkship, graduate students received the Clinical Performance Examination (CPX) training, which utilized SPs who complained of symptoms and signs of diseases. CPX training allows for the examination of one case for 40 min. One student takes 10 min to complete the following process with a SP: query the SP volunteer, and conduct the four aspects of TCM diagnosis and physical examinations as required. When the SP volunteers leave, the student explains the suspected disease and the diagnosis plan to the instructor, and then the instructor and SP trainer provide feedback. To minimize bias, all training sessions are conducted by the same instructor.

The instructors had previous experience using role-play as a teaching method and the SSPs familiar with the scripts through live group situational rehearsal. During the whole process, SSPs could timely record and feedback instructors' opinions on the simulation training, such as the level of difficulty, along with the appropriateness and ambiguity. Finally, the scripts were revised and finalized based on the results of the item of real-time training analysis and the cognitive debriefing in order to ensure comprehensibility and comprehensiveness. The instructors further explained the key points of imitation of various diseases. Before starting the curriculum in 2018, TCM professionals would establish a case database that met the scripts’ requirements, and different cases selection would be updated every year.
